# Measuring the Success of Community Science: The Northern California Household Exposure Study

**DOI:** 10.1289/ehp.1103734

**Published:** 2011-12-06

**Authors:** Phil Brown, Julia Green Brody, Rachel Morello-Frosch, Jessica Tovar, Ami R. Zota, Ruthann A. Rudel

**Affiliations:** 1 Department of Sociology and Center for Environmental Studies, Brown University, Providence, Rhode Island, USA; 2 Silent Spring Institute, Newton, Massachusetts, USA; 3 School of Public Health and Department of Environmental Science, Policy and Management, University of California–Berkeley, Berkeley, California, USA; 4 Communities for a Better Environment, Oakland, California, USA; 5 Program on Reproductive Health and the Environment, University of California–San Francisco, Oakland, California, USA

**Keywords:** breast cancer, community-based participatory research, environmental justice, evaluation metrics, exposure science

## Abstract

Background: Environmental health research involving community participation has increased substantially since the National Institute of Environmental Health Sciences (NIEHS) environmental justice and community-based participatory research (CBPR) partnerships began in the mid-1990s. The goals of these partnerships are to inform and empower better decisions about exposures, foster trust, and generate scientific knowledge to reduce environmental health disparities in low-income, minority communities. Peer-reviewed publication and clinical health outcomes alone are inadequate criteria to judge the success of projects in meeting these goals; therefore, new strategies for evaluating success are needed.

Objectives: We reviewed the methods used to evaluate our project, “Linking Breast Cancer Advocacy and Environmental Justice,” to help identify successful CBPR methods and to assist other teams in documenting effectiveness. Although our project precedes the development of the NIEHS Evaluation Metrics Manual, a schema to evaluate the success of projects funded through the Partnerships in Environmental Public Health (PEPH), our work reported here illustrates the record keeping and self-reflection anticipated in NIEHS’s PEPH.

Discussion: Evaluation strategies should assess how CBPR partnerships meet the goals of all partners. Our partnership, which included two strong community-based organizations, produced a team that helped all partners gain organizational capacity. Environmental sampling in homes and reporting the results of that effort had community education and constituency-building benefits. Scientific results contributed to a court decision that required cumulative impact assessment for an oil refinery and to new policies for chemicals used in consumer products. All partners leveraged additional funding to extend their work.

Conclusions: An appropriate evaluation strategy can demonstrate how CBPR projects can advance science, support community empowerment, increase environmental health literacy, and generate individual and policy action to protect health.

Community-based participatory research (CBPR) is an approach to academic–community partnerships that shares power with community partners in all aspects of the research process and benefits communities through interventions or policy change. CBPR projects increase community engagement in research to generate scientific knowledge, improve public trust and understanding of environmental health science, inform culturally and socially appropriate intervention methods, improve public health decisions and stimulate action, and contribute to environmental justice (EJ) (Minkler et al. 2008; O’Fallon and Dearry 2002). Grants supporting research that involves community participation have increased dramatically and gained in academic respectability since 1996, when the National Institute of Environmental Health Sciences (NIEHS) started funding such research (Wolfson and Parries 2010), which, in turn, led to increasing pressure to more precisely evaluate outcomes (Drew et al. 2010).

Evaluating CBPR success can be difficult, because peer-reviewed publication and clinical health outcomes alone are inadequate criteria and even tangential to many community partnerships. A recent contribution to CBPR evaluation extracted outcomes from grantee reports (Baron et al. 2009), but the information accessible in the reports was limited because the evaluation metrics were new and research teams had not yet consistently implemented them. In addition, these reports focused on successful outcomes, omitting process factors, challenges, and failures.

To help stimulate other teams to think more thoroughly and expansively about the outcomes of their CBPR work, we report here on the northern California Household Exposure Study (HES), which was funded through a grant titled “Linking Breast Cancer Advocacy and Environmental Justice” under NIEHS’s EJ program (Silent Spring Institute 2011b). We illustrate how projects of this type can be assessed with respect to environmental health advocacy and community-building outcomes. Partners in the HES were Silent Spring Institute, which focuses on the environment, breast cancer, and women’s health; Communities for a Better Environment (CBE 2008), an EJ organization in California; and faculty at Brown University and the University of California, Berkeley. EJ organizations are self-defined groups committed to focusing on racial and social class inequalities in both exposure to environmental problems and access to environmental benefits. Our project was an extension of the Silent Spring Institute HES, which started in Cape Cod, Massachusetts, in 1999 (Silent Spring Institute 2011a). In 2004–2009, we conducted an exposure study in two neighborhoods bordering an oil refinery in Richmond, California, and a rural neighborhood in Bolinas, California, that served as a regional comparison area (Brody et al. 2009). Our scientific goal was to characterize cumulative impact in an EJ community, which refers to an area of low-income or ethnic minority residents who are disproportionately affected by environmental pollution [U.S. Environmental Protection Agency (EPA) 1994], by measuring numerous pollutants from outdoor and indoor sources and to assess differences compared with homes in non-EJ communities. The U.S. EPA defines an EJ community by calculating (on a regional basis) the percent of the census block group that is minority and the percent of the block group that is low-income (less than twice the federal poverty level) (U.S. EPA 2011). If the combined demographics make the block group high enough to rank in the top 15% of block groups, it is an EJ community (Rhode Island Department of Environmental Management 2009). Activists usually have a broader definition that emphasizes high cumulative exposure in minority and poor areas.

Our policy goals were to inform local decisions about the oil refinery, state policies regarding chemicals, and national decisions about endocrine-disrupting compounds (EDCs) in consumer products. Our educational goals were to inform community members about determinants of their indoor and outdoor air quality, strategies for exposure reduction, and potential implications for health.

## Methods

Data to assess the project’s production of new science included the number of articles published and conferences and workshops where we were invited to speak. To gauge overall community support, we collected written evaluation forms after each community meeting, had two team members take detailed notes of questions and responses, and held team debriefings immediately afterward. To assess participants’ understanding of the overall study and their data, we interviewed them before sampling and after results were returned. We examined team process by group phone calls at least monthly. To assess overall project process and success, we conducted advisory board meetings at which we recorded notes and for which we interviewed board members, conducted team interviews of each collaborating organization, and analyzed government decisions, court decisions, and media coverage. We also reviewed other evaluation studies of NIEHS programs.

We focused our self-evaluation on the following questions, which are discussed in detail below: 1) How did the project influence participation by affected constituencies? 2) Did CBPR practices influence the research design? 3) Did research practices (e.g., data collection) contribute to community education or engagement? 4) Did research needs conflict with community needs? How was this resolved? 5) Did the project advance theory or methods, as well as producing research results? 6) How did project practices contribute to durable team building? 7) Did the project contribute to financial sustainability for environmental health work? 8) Did scientific results lead to action? 9) Have other teams adopted project methods or built on results? 10) Did researchers, including students, continue to pursue the topics addressed by the project?

## Results and Discussion

We first address elements of the project’s collaborative process (evaluation questions 1–4 and 6 above) and then summarize the scientific contributions and benefits of our project to the academic partners (questions 2, 5, and 10), to community-based organizations (CBOs; questions 6–8), to the communities where these organizations work (question 8), and to the broader environmental health community and society at large (questions 5, 9, and 10). Finally, we summarize criteria of effectiveness. Although we emphasize our successes, our intent is to illustrate what other teams should take note of, so they can make a case for their effectiveness.

*Elements of collaboration.* Two types of CBOs. To understand collaboration in CBPR, it is necessary to examine the structure, focus, and constituency of the CBOs involved in the research. CBE is an EJ organization that does community organizing and legal work. Like other CBOs, CBE has responded to the increasing need to provide scientific evidence to support advocacy by adding scientists to its staff, typically to provide expertise in secondary data analysis and assist in collaborations with academics.

In contrast, the Silent Spring Institute, which was formed by a community of women to address concerns about the high incidence of breast cancer on Cape Cod and the potential environmental causes, is a CBO that conducts primary scientific research. The breast cancer activists who founded Silent Spring believed they needed to define and carry out research in “a lab of our own,” because academics were not pursuing environmental causation (Brown et al. 2006). Initial support for the institute was in Massachusetts, but the implications of the institute’s research on EDCs have expanded support to include a network of environmental breast cancer activists throughout the United States (McCormick et al. 2003).

Advisory council. Much of the CBPR literature points to the important role of advisory boards in guiding research collaboratives to ensure that the interests of both the CBOs and the community remain paramount (Israel et al. 1998). We had advisory councils on each coast because there were local issues for the two sites—Cape Cod and northern California. The northern California council comprised leaders of four CBOs (Breast Cancer Action, Breast Cancer Fund, Commonweal, and West County Toxics Coalition), two Richmond residents, one environmental health scientist, and one state public health official. The Massachusetts council comprised leaders of four CBOs (Boston Urban Asthma Coalition, Massachusetts Breast Cancer Coalition, Massachusetts Coalition for Occupational Safety and Health, and Toxics Action Center), two physicians, and a nurse. The councils met annually, and we consulted members between meetings. As data collection shifted to California, the Massachusetts council met less frequently. The California council played a major role by suggesting we add the Bolinas site. The advisory councils also served the project’s aims by introducing EJ and breast cancer activists to each other and by providing opportunities to deepen understanding of each other’s work.

The totality of collaboration. Our initial sampling plan was to randomly select 50 residents of two Richmond neighborhoods. When the California advisory council members asked for a regional comparison group, we changed the plan to include 40 Richmond and 10 Bolinas residents. We conducted educational meetings and canvasing in Richmond to explain and generate interest in the study, and many community residents volunteered to participate. Therefore, to balance the need for a representative study sample with the goal of building a supportive community partnership, we allowed 20 slots for Richmond volunteers and preserved 20 randomly selected Richmond slots.

Building team community. We regularly convened research partners through bimonthly (sometimes weekly) conference calls, yearly in-person meetings (2–3 days), visits for scientific training, e-mail, and visits when people were traveling for other reasons. Phone calls also included discussions of upcoming cultural activities, reports on the latest achievements of other projects, and discussion of activities by related community groups. Although time-consuming, these discussions enhanced mutual understanding about different organizational cultures and indicated who needed additional help because of the demands of activities outside the project. The team took advantage of in-person meetings as an occasion for social gatherings, which further promoted personal relationships.

Despite our informal working relationships the collaborative drafted a formal written agreement regarding data management, publications and authorship, and decision-making processes. These types of memoranda of understanding have helped clarify division of labor, roles, and responsibilities among partners in other CBPR projects (e.g., Israel et al. 1998). Even when academic partners concur with such agreements, they may face challenges in getting their institutions to allow them to sign on. For example, Brown University’s Office of Sponsored Projects initially balked at Silent Spring Institute’s authorship agreement, which gave all investigators the right to use data from the project after consultation with each other, as opposed to Brown controlling publication decisions.

Need to be frank about problems. The success of any collaboration is dependent upon the ability of partners to address challenges that emerge as the projects progress. In fact, it is critical that collaborators openly discuss problems as they arise.

For example, CBE had concerns about the demands of the research project on staff time, and its potential to move the organization away from its core mission. At times, CBE needed to expend virtually all its staff efforts on organizing around the Richmond refinery. We resolved this tension by adjusting our recruitment timeline and adding local student staffing. Moreover, CBE found that its door-to-door canvasing for recruitment and data collection enhanced community-organizing efforts. Ultimately, CBE managed to collect all the household air and dust samples on time, which was scientifically important to reduce interference of seasonal weather.

Silent Spring had similar concerns about going off-mission, because some aspects of the research, such as studying metals associated with the refinery, were not specifically related to women’s health. However, the team concluded that the benefits of this research to community partners justified the organization’s participation. In addition, Silent Spring successfully sought additional funding for the Massachusetts portion of the study, so that more resources could be devoted to studying industrial pollutants in Richmond.

Ongoing collaboration. Although the end of the NIEHS-funded project concluded our formal 4-year research collaboration, the team secured new funding from the Avon Foundation for Women to conduct a community environmental health survey about local concerns related to neighborhood conditions, health problems, and health care access. This helped the project use study results to support residents’ advocacy efforts and research dissemination (Cohen et al. 2011). CBE has continued to expand its outdoor air monitoring in other locations, such as East Oakland and Southern California (CBE 2008; Our Blog: News from the Frontlines 2011).

The Brown–Berkeley–Silent Spring collaboration continued with two National Science Foundation grants on biomonitoring and one on flame retardants and an R01 grant on ethical and legal challenges in communicating personal exposure results, and Brown University’s new Formative Children’s Environmental Health Center includes Silent Spring as a partner. In total, the team got seven related grants [for a list of grants, see Supplemental Material, Table 1 (http://dx.doi.org/10.1289/ehp.1103734)].

*Benefits of our project to collaborators and communities.* Benefits to science. We designed scientific questions to meet the needs of each study partner. We briefly review here the resulting scientific contributions, referring readers to our scientific publications [for a list of publications, see Supplemental Material, Table 2 (http://dx.doi.org/10.1289/ehp.1103734)].

Silent Spring Institute’s HES on Cape Cod was the beginning of extensive work on household exposure to EDCs, including the first indoor measures for 30 compounds (Rudel et al. 2003). The study identified a wood floor finish as a widespread ongoing source of polychlorinated biphenyls (Rudel et al. 2008). Expanding the HES to California allowed us to examine exposure in a poor, minority community. In particular, we showed the indoor penetration of chemicals from heavy oil combustion and documented that the disproportionate cumulative impact of pollution in Richmond was more pronounced inside homes than outdoors (Brody et al. 2009). Results showed that products used indoors were the major source for EDCs in air (Rudel et al. 2010). Having found high polybrominated diphenyl ether (PBDE) levels in Massachusetts, we tested for them in California and recorded some of the highest levels of in-home dust ever found (Zota et al. 2008), a finding linked to California’s strict flammability standard for furniture foam (State of California 2000).

Another component of our project focused on reporting individual results to study participants and studywide results to community members (“report-back”). We offered all individuals the opportunity to receive their results, and all but two accepted. We developed new tools for these report-back activities, and we studied the effects (Brody et al. 2007). Reporting results has been controversial because researchers worry about reporting information with uncertain health or intervention implications, but participants often want their results, and they have become a tool for public health advocacy (Curtis and Wilding 2007). Some institutional review boards (IRBs) do not allow report-back, and part of our success was convincing the Brown IRB to approve it. By interviewing participants after they received their results, we found that individual report-back contributed to environmental health education and stimulated behavior change and public involvement, and we found no evidence of harm from undue stress (Altman et al. 2008). Participants learned that numerous EDCs from common products were in household air and dust and in urine samples; banned substances, such as DDT (dichlorodiphenyltrichloroethane) are detected today; and many household chemicals are unregulated and unstudied. In Richmond and Bolinas, we found differences in expectations and interpretations about exposures. Richmond residents expected pollutants from industry; Bolinas participants, living in a rural area, expected “a clean bill of health” (Adams et al. 2011). Because of our experience with report-back approaches, including our evaluation of effects on participants, our team was asked to consult with other researchers and government agencies, and several studies have adopted models similar to ours.

Benefits to academic partners. For many, academic life has an ethical component that values research translation for human betterment. Collaboration with community partners helps researchers fill this role and parallels the “research altruism” we found among research participants who offered their homes and time to advance scientific knowledge. For academics, there is some risk in CBPR: It is complex, demands more time to develop collaborative relationships, and opens researchers to criticism that they are too applied and perhaps even biased. Yet CBPR practitioners can be leaders as scientists who are seen as honest, accountable, and supportive of community needs. This strengthens their ability to conduct research and reflects well on their university in its community relations.

Academic partners benefited from excellent data to work with and publish, as demonstrated by publications, presentations at scientific and governmental conferences, and being sought out by scientists and government agencies doing exposure work, including the California state biomonitoring program. Both academic partners received major National Science Foundation grants that were direct outcomes of the project. The academics also gained by having high-quality opportunities to train students in CBPR. Five students got research and collaboration experience and publications in highly cited journals [see Supplemental Material, Table 2 (http://dx.doi.org/10.1289/ehp.1103734)], and two dissertations were written. Our project led us to run workshops on CBPR for faculty and graduate students and one of us to gain a university-funded postdoctoral position in environmental ethics.

Benefits to Silent Spring Institute. The fact that Silent Spring Institute received a competitive NIEHS grant added to its visibility and stature as a research organization. Federal funding signals to various reviewers that Silent Spring Institute’s science is innovative and technically credible. Annual NIEHS grantee meetings also created opportunities to meet other EJ and academic researchers. Silent Spring researchers were invited to serve on grant reviews, which provided insight into current methods and research agendas and introduced Silent Spring to a wider range of potential collaborators.

For Silent Spring’s breast cancer activist constituency, the California HES provided a valuable comparison with Cape Cod. It also helped expand the diversity of breast cancer activism. The partnership with Brown University offered the benefit of a social scientist as an external “evaluator” who helped the Silent Spring Institute board to better understand organizational challenges. Adjunct appointments at Brown gave access to the university electronic library and brought credibility and contact with other researchers.

Benefits to CBE. CBE gained stronger organizing, because they went door to door to recruit participants and also had occasions to bring large groups together in community meetings to prepare for the HES and report results. Neighborhood leaders made their neighborhood center available to CBE for meetings for the first time; previous requests had been turned down. Ultimately, the project gave CBE data about specific refinery emissions and cumulative impact for its advocacy campaign.

CBE’s standing with community members and elected officials was strengthened because they brought science to the community. The research was empowering for residents, who brought their results to public hearings. It offered good leadership development by training CBE staff in research methods and interpretation and giving residents opportunities to discuss and testify about results. City planning officials asked the team to submit testimony on a refinery expansion proposal. Individuals who received their own results and completed the HES questionnaire [see Supplemental Material (http://dx.doi.org/10.1289/ehp.1103734)] gained knowledge about their exposure to chemicals in and outside the home, which helped them consider personal exposure reduction practices as well as communitywide changes.

Although CBE’s initial focus was on community exposures to pollutants from the Richmond oil refinery, it became clear that demonstrating cumulative impacts of multiple pollutant exposures was also relevant to the organization’s mission. CBE, with help from Silent Spring, received a grant from the Avon Foundation for Women (2011) to conduct a health survey in Richmond with a larger sample (198 respondents provided health data on 722 individuals) than the HES. This helped CBE address community members’ requests for such a project and yielded additional data to show disproportionate health impact on Richmond residents.

Benefits to communities. Richmond gained as a community. In terms of process, there was a strong sense of project “ownership” in Richmond. Residents expressed this ownership at a very early phase by volunteering to participate, and later by active participation at community meetings and public hearings. People were eager to use data for local needs, and they also put forth additional research questions, as in the case of a woman who suggested that we study another industrial community for comparison. Another person suggested using the Environmental Impact Report (City of Richmond 2008), a complex document that analyzes the environmental effects associated with a proposed development project in accordance with the California Environmental Quality Act of 1970 (State of California 1970) to see which pollutants were predicted to rise with refinery expansion, and then see if those were the same chemicals we detected. Another said we should look at chemicals that are now at or near the standards set by the U.S. EPA (2011) and the California Environmental Protection Agency (2011) in order to point out that the expansion would likely lead to exceedances. These are examples of hoped-for future research, as well as suggestions for how to apply results from our project. In this way, people were now using their own data. Project scientists were not treated as distant experts presenting material but as part of a team with the local organizers.

Our observations of meetings and written evaluation forms indicated a changed public perception of science. Residents said the project demystified science by bringing it into people’s homes, especially because the sampling was conducted by CBE staff who were familiar to them. It also changed ideas about who conducts science by having women and people of color present scientific results at community meetings in Spanish and in English. In terms of outcomes, the data helped elect new town councilors who opposed refinery expansion, gave Richmond residents sufficient information to convince city government to deny refinery expansion, and helped them win a lawsuit (Brody et al. 2009).

## Criteria of Effectiveness

CBPR projects can meet multiple criteria of effectiveness, and all should not be expected to achieve the same set of outcomes. Here we address important criteria in which we have succeeded. Because we recapitulate items mentioned earlier, our points are brief illustrations [criteria and examples are summarized in Supplemental Material, Table 3 (http://dx.doi.org/10.1289/ehp.1103734)]. Again, our intent is to point out outcomes in order to stimulate others to notice and record effects of their own CBPR projects.

*Production of new science.* Publication in highly cited journals is an appropriate CBPR effectiveness measure, because it builds a credible basis for public health action and informs others about project outcomes. Our publications spanned our disciplines of sociology, environmental science, and public health (Adams et al. 2011; Altman et al. 2008; Brody et al. 2007, 2009; Brown et al. 2010; Morello-Frosch et al. 2009; Rudel et al. 2010; Zota et al. 2008).

We made theoretical advances by developing three new concepts. The “research right-to-know” concept holds that individuals and communities have the right to know the results of research conducted on them and their surroundings, especially in a context that supports autonomy and action (Brody et al. 2007; Morello-Frosch et al. 2009). The concept of “exposure experience,” based on the “illness experience” approach in medical sociology and anthropology (Rier 2010), explains how people take in data about their own exposures, including an understanding of absolute and relative exposures, attributions of sources and blame, perceived harm and worry, and opportunities to ameliorate conditions (Adams et al. 2011; Altman et al. 2008). Our concept of “research altruism” explains the willingness of people to participate in research. Many participants reported that they agreed to participate because they felt that trusted organizations were doing good work that would benefit others (Adams et al. 2011; Altman et al. 2008). In addition to the general bioethical concept of altruism to unknown recipients, for example, in blood donation (Titmuss 1972), “research altruism” adds a benefit for known persons (family, friends, neighbors) and a supportive attitude toward the organization doing the research.

We made methodological advances. Our exposure science contributed to the conceptualization of cumulative impact in indoor environments as an EJ issue (Brody et al. 2009), the use of geographic and demographic analysis to evaluate exposure consequences of public policies (Zota et al. 2008), and the study of chemical mixtures (Rudel et al. 2010). Our paired indoor–outdoor sampling demonstrated that outdoor contaminants (vanadium, nickel, particulate matter) build up inside homes. We showed the value of analyzing a very wide range of compounds to take into account both outdoor and indoor sources of exposure, including consumer products. To our knowledge, we developed the most comprehensive and participatory approach to report-back of biomonitoring and household exposure to date. Further, we designed an innovative manner of presentation with graphs designed for laypeople, and through our interviews, we evaluated how well the presentations were understood.

*How science benefits community members.* CBPR projects should support the advocacy needs of CBOs to generate health-protective action, and this project was effective in that realm. CBE presented data to the Richmond City Council and Planning Commission (City of Richmond 2008, 2011) and argued in court for cumulative impact assessments to be included in oil refinery permit applications, an approach central to our research. The advocacy that resulted in a legal victory that blocked refinery expansion may be considered a public health intervention. At the state and national levels, Silent Spring Institute’s allies in breast cancer and environmental organizations used results to support chemicals policy reform and consumer movements. Further, our interviews found that report-back itself is a form of intervention that educated individuals and communities, led to personal changes in household practices, and increased civic participation (Altman et al. 2008). At a larger scale of benefits to the broader community, findings were used by a state legislator to seek removal of the California furniture flammability standard. Our data were used in other legislation as well, such as a failed California bill (Senate Bill 772; Consumer Federation of California 2009) to ban halogenated flame retardants in children’s products. Our results also supported the efforts of breast cancer activists to win reformulation of cosmetics, cleaners, and other consumer products.

*Relationship between partners and its effects on public health.* As mentioned in earlier sections, this project’s unique collaboration of partners was successful in a major intervention to reduce future contaminant emissions by limiting the refinery. This was possible because of open, frank, and continually evolving discussion among partners and by frequent self-evaluation and reflexive analysis of our interactions. Rather than focus on flaws that one partner might see in another’s involvement at a given time, we emphasized positive solutions to what we considered temporary difficulties.

*Effect of communication.* Media attention can broadly disseminate research findings and aid in advocacy and intervention. Our work was reported in many print and online outlets, including the *Wall Street Journal*, *Sacramento Bee*, *National Geographic*, Reuters, and the *Los Angeles Times*. *Consumer Reports* (2005) used our findings to advise people on safer products [see Supplemental Material, Table 4 (http://dx.doi.org/10.1289/ehp.1103734) for examples of the extensive media coverage]. This coverage resulted from our proactive efforts to hone messages, with help from the respected environmental health media organization Science Communication Network (2011), to develop press lists, issue releases, and to be accessible to reporters.

We always viewed our project as dealing with the larger interface of EJ and breast cancer advocacy, not just the specific tasks of our Richmond work. We tailored our communication activities to this by convening meetings on EJ–breast cancer connections with other EJ groups. We strove to educate breast cancer activists about EJ and also put much effort into educating EJ groups about emerging contaminants.

Because the affected community should be the primary recipient of knowledge from our projects, we reported results to study participants and community members before scientific publication. Individual report-back was built into the project as a core responsibility; we asked participants during the informed consent whether they wanted to receive their own results, and nearly all did. We held annual community meetings to present and discuss plans, progress, and results and many smaller meetings with community groups and officials. Our goals were to seek input, provide data to community members they could use personally and for advocacy, and inform public officials. Reporting individual results stimulated participation in community meetings, and the meeting format with presentations and small-group discussions in English and Spanish led to an action focus. Because our interviews with participants show that report-back increases environmental health literacy and stimulates personal and civic action, disseminating these methods has public health impact (Adams et al. 2011).

*Contributing to public participation.* We contributed to public participation in science by advancing CBPR methods and ethics. We achieved an extremely high degree of community engagement and science education, not only with CBO partners but also with study participants and nonparticipant residents. In responding to community advisory board suggestions, we included a route for community input to the actual science, through choice of compounds to analyze and through sampling frame. Our individual and community-level report-back gave CBOs and local residents much power over the use of knowledge. We put much effort into getting the Brown IRB to allow individual report-back, which is still not common.

## Conclusion

Our case study illustrates evaluation of a CBPR project, using criteria that better capture the types of outcomes that are relevant to this field. Our outcomes include scientific results, methods and theory development, advocacy applications, broad dissemination, adoption of our approaches by others, benefits to collaborators and communities, and support for the breast cancer and EJ movements. We believe this provides support for expanded CBPR and EJ funding and for training of faculty, students, and community members in CBPR.

We recommend that more CBOs take on the capacity-building challenge of being CBPR principal investigators and that more CBOs develop science expertise in order to pursue primary research to address their constituencies’ needs. We urge that community advisory boards be used to help shape research design, not merely to oversee community collaboration issues. We recommend extensive formal and informal interactions between partners, including frank discussion of responsibilities, which builds a strong team and allows for adjustments when some components go wrong. We especially value the continuity of collaborations beyond a single large project and encourage all partners to constantly search for funded and nonfunded opportunities to continue their partnership.

Based on our experience, we believe that CBPR partnerships can strengthen this field by having partners talk to each other about how their collaboration is going. These evaluations should consider the multiple scientific, educational, policy, community engagement, and capacity-building goals of a project in relationship to benefits and stresses for each partner. Considering these issues frequently during the course of a project will both collect ongoing evaluation data and stimulate changes to address problems. Further, CBPR evaluations should assess effects on the broader community and on environmental health science. The 10 questions we list in “Methods” offer guidance for such evaluation. Funding for CBPR projects should include the development of such evaluation approaches, which include but go beyond traditional evaluation metrics. Successful evaluation is critical to making the case for continued support of CBPR, and CBPR practitioners need to take the initiative to create evaluation programs that address the strengths of CBPR approaches.

## Supplemental Material

(188 KB) PDF
