# Supplementary material for: Measuring the Success of Community Science: The Northern California Household Exposure Study
Source: Environ Health Perspect. 2011 Dec 6;120(3):326–31. doi: 10.1289/ehp.1103734 (PMC3295345; doi:10.1289/ehp.1103734)
Supplement: (188 KB) PDF [file ehp.1103734.s001.pdf]

Supplemental Material

MEASURING THE SUCCESS OF COMMUNITY SCIENCE: THE NORTHERN CALIFORNIA HOUSEHOLD EXPOSURE STUDY

[1]Phil Brown, [2]Julia Green Brody, [3]Rachel Morello-Frosch, [4]Jessica Tovar, [5]Ami R. Zota, and [2]Ruthann A. Rudel

[1]Brown University, Department of Sociology and Center for Environmental Studies, Box 1916, Providence RI 02912, USA.

[2]Silent Spring Institute, 29 Crafts Street, Newton MA 02458, USA

[3]University of California, Berkeley, School of Public Health and Department of Environmental Science, Policy and Management, 137 Mulford Hall, Berkeley CA 94720-3114, USA.

[4] Communities for a Better Environment, 1904 Franklin Street, Suite 600
Oakland, CA 94612

[5]Program on Reproductive Health and the Environment, University of California San Francisco, 1330 Broadway, Suite 1100, Oakland, CA 94612

Corresponding author address:
Phil Brown, Brown University, Department of Sociology and Center for Environmental Studies, Box 1916, Providence RI 02912, USA. Email: phil_brown@brown.edu. Tel: 401-863-2633. Fax: 410-863-3213

Supplemental Material Contents

- Supplemental Material, Table 1 – Additional Grants Resulting from Breast Cancer and EJ Household Exposure Study Grant……………………………………………………..page 1
- Supplemental Material, Table 2 – Publications………………………...……………page 3
- Supplemental Material, Table 3 –Criteria of Effectiveness……………………………page 5
- Supplemental Material, Table 4 – Examples of News Media Coverage………………..page 6
- Household Exposure Study Survey…………………………………………….………page 8

Supplemental Material, Table 1 – Additional Grants Resulting from the Breast Cancer and EJ Household Exposure Study Grant

Grant Title	Funding Agency
"The 'Research Right-to-Know': Ethics and Values in Communicating Research Data to Individuals and Communities"	National Science Foundation: $300,000, 2005-2008 (PI Julia Brody, SES 0450837)
Partnerships in Environmental Public Health supplement to "Linking Breast Cancer Advocacy and Environment Justice"	National Institute of Environmental Health Sciences: $139,805, 2008-2009 (PI Julia Brody, R25 ES013258)
"Toxic Ignorance and the New Right-to-Know: The Implications of Biomonitoring for Regulatory Science."	National Science Foundation: $407539, 2009-2012 (PI Rachel Morello-Frosch)
"Flame Retardant Chemicals: Their Social Discovery as a Case Study for Emerging Contaminants"	National Science Foundation: $432,676, 2009-2012 (PI Phil Brown)
"Ethical and Legal Challenges in Communicating Biomonitoring and Personal Exposure Results to Participants"	National Institutes of Health: $1,826,012, 2009-2014 (PI Julia Brody, R01ES017514-02)
"Richmond Community Health Survey"	Avon Foundation: $125,000
"Pollution at Home: Exposures in a Fence-line Environmental Justice Community"	New York Community Trust: $75,000, 2009-2010 (PI Julia Brody)

Supplemental Material, Table 2 – Publications

Title (Authors)	Journal
"Improving Disclosure and Consent: Is It Safe? New Ethics for Reporting Personal Exposures to Environmental Chemicals" (Julia Green Brody, Rachel Morello-Frosch, Phil Brown, Ruthann A. Rudel, Rebecca Gasior Altman, Margaret Frye, Cheryl C. Osimo, Carla Perez, and Liesel M. Seryak)	*American Journal of Public Health* 2007 97: 1547-1554.
"Pollution Comes Home and Gets Personal: Women's Experience of Household Toxic Exposure" (Rebecca Altman, Julia Green Brody, Ruthann A. Rudel, Rachel Morello-Frosch, Phil Brown, and Mara Averick)	*Journal of Health and Social Behavior* 2008 49:417-435.
"Elevated House Dust and Serum Concentrations of PBDEs in California: Unintended Consequences of Furniture Flammability Standards?" (Ami Zota , Ruthann Rudel, Rachel Morello-Frosch, and Julia Brody)	*Environmental Science & Technology* 2008:42(21):8158-8164.
"'Toxic Ignorance' and the Right-to-Know: Assessing Strategies for Biomonitoring Results Communication in a Survey of Scientists and Study Participants" (Rachel Morello-Frosch, Julia Green Brody, Phil Brown, Rebecca Gasior Altman, Ruthann A. Rudel, Carla Pérez)	*Environmental Health* 2009 8:6.
"Linking Exposure Assessment Science with Policy Objectives for Environmental Justice and Breast Cancer Advocacy: The Northern California Household Exposure Study" (Julia Green Brody, Rachel Morello-Frosch, Ami Zota, Phil Brown, Carla Pérez, and Ruthann A. Rudel)	*American Journal of Public Health* 2009 99:S600-S609
"Endocrine disrupting chemicals in indoor and outdoor air" (Ruthann A. Rudel and Laura J. Perovich)	*Atmospheric Environment* ***2009*** 43: 170-181.
"Semivolatile Endocrine Disrupting Compounds in Paired Indoor and Outdoor Air in Two Northern California Communities" (Ruthann A. Rudel, Robin E. Dodson, Laura J. Perovich, Rachel Morello-Frosch, David E. Camann, Michelle M. Zuniga, Alice Y. Yau, Allan C. Just, and Julia Green Brody	*Environmental Science & Technology,* 2010 44 (17), pp 6583–6590
"Institutional Review Board Challenges Related to Community-Based Participatory Research on Human Exposure to Environmental Toxins: A Case Study" (Phil Brown, Rachel Morello-Frosch, Julia Green Brody, Rebecca Gasior Altman, Ruthann A. Rudel, Laura Senier, Carla Pérez and Ruth Simpson)	*Environmental Health* 2010 9:39.
"Disentangling the Exposure Experience: The Roles of Community Context and Report-back of Environmental Exposure Data" (Crystal Adams, Phil Brown, Rachel Morello-Frosch, Julia Green Brody, Ruthann Rudel, Ami Zota, Sarah Dunagan, Jessica Tovar, and Sharyle Patton)	*Journal of Health and Social Behavior* 2011 52 (2):180-196.

"Public Sociology for Environmental Health and Environmental Justice" (Alissa Cordner, Alison Cohen, and Phil Brown)	Pp. 97-106 in Philip Nyden, Leslie Hossfeld, and Gendolyn Nyden, eds. Public Sociology; Research, Action, and Change. Los Angeles: Sage.
"Our Environment, Our Health: A Community-Based Participatory Environmental Health Survey in Richmond, California" (Alison Cohen, Andrea Lopez, Nile Malloy and Rachel Morello-Frosch)	*Health Education and Behavior* published online 8 July 2011
"Lessons Learned from Flame Retardant Use and Regulation Could Enhance Future Control of Potentially Hazardous Chemicals" (Phil Brown and Alissa Cordner)	*Health Affairs* 2011 30 (5):1-9.

Supplemental Material, Table 3 –Criteria of Effectiveness and Examples

Type of Output	Examples
Theory development	• Research right-to-know<br>• Exposure experience<br>• Research altruism
Methods	• Innovative approaches to report-back of personal exposure data.<br>• Extensive analysis of cumulative impact of outdoor sources and indoor sources, including novel analytes and analysis of mixtures<br>• Geographic and demographic analysis of consequences of flammability policies
Broad dissemination	Publications and conference presentations; discussion of our work in various environmental health and environmental justice circles; news media coverage
Advocacy applications	• Increased community engagement.<br>• Evidence for CBE to use with Richmond Planning Commissions and in litigation in successful effort to block refinery expansion.<br>• Evidence in support of efforts to change flammability standards.<br>• Evidence is support of new policies and consumer choices for chemicals in consumer products.<br>• Increased environmental health literacy and motivation to reduce exposure after report-back
Adoption of approaches by others	Consultation and implementation of report-back of personal exposure data to participants in the Breast Cancer and the Environment Research Centers and California Biomonitoring Program.
Support for the breast cancer and environmental justice movements	Meetings and educational forums that bridge the two movements: breast cancer “interest group” session at NIEHS environmental justice grantee meetings; aid to West Harlem Environmental Action (WEACT) in organizing first conference in US on breast cancer, women of color, and the environment; encourage breast cancer movement to adopt an environmental justice perspective
Benefits to collaborators and communities	Publications, conference presentations, additional grants (as noted in tables 1 and 2 of Supplemental Material; skill- and knowledge-building

Supplemental Material, Table 4 – Examples of News Media Coverage

WebMD, "Endocrine-disrupting chemicals are indoor risk," August 5, 2010, By Kathleen Doheny http://www.webmd.com/lung/news/20100805/endocrine-disrupting-chemicals-are-indoor-risk

UPI, "High endocrine disruptor levels in homes,"August 5, 2010 http://www.upi.com/Health_News/2010/08/05/High-endocrine-disruptor-levels-in-homes/UPI-52591281055997/

Reuters, "Pollution at home often lurks unrecognized," December 26, 2008, By, Amy Norton http://silentspring.org/news/media-coverage/reuters-pollution-home-often-lurks-unrecognized

Los Angeles Times, "Did the state kill my cat?" October 17, 2008, Op ed By Arlene Blum

Capitol Weekly, "Leno asks Governor to ban controversial fire retardants by executive order," October 7, 2008 By, Malcolm Maclachlan http://www.capitolweekly.net/article.php?_adctlid=v|jq2q43wvsl855o|xgklia7hkp9q7e&issueId=xg5kpub17clp0y&xid=xgk1477grhxq7b

Los Angeles Times, "Californians have much higher levels of flame-retardant PBDE in their blood, October 3, 2008, By Thomas H. Maugh II http://articles.latimes.com/2008/oct/04/science/sci-pbde4

The Sacramento Bee, "Fire retardants showing up in blood, scientists report," October 2, 2008, By, Chris Bowman

The Wall Street Journal Market Watch, "Californians Have Toxic Flame Retardants in Their Blood at Levels Twice the National Average," October 1, 2008

Bay City News, "Study: Refinery pollution trapped in homes," April 09, 2008 http://abclocal.go.com/kgo/story?section=news/local&id=6070514

National Geographic, "Chemicals Within Us," October 2006, By David Ewing Duncan http://ngm.nationalgeographic.com/2006/10/toxic-people/duncan-text

The New York Times, "New Emission Rule for Bay Area Refineries," July 21, 2005, By Carolyn Marshall http://www.nytimes.com/2005/07/21/national/21refineries.html?_r=1&adxnnl=1&oref=slogin&adxnnlx=1311795137-Ld/IjOwdHbzaYlxvM5x1BA

Consumer Reports on Health, "10 Ways to Reduce Your Exposure to Chemicals," April 2005

HOUSEHOLD EXPOSURE STUDY SURVEY

Interviewer check: ID number: ________ Date: ________ Time: ____:____

I will be asking you a number of questions about your household that will help us interpret the air and dust samples we collected. I need to read the questions exactly as they are written. Some questions may sound long—please bear with me. You may ask me to repeat any question or to explain any part of it that you do not understand. First I'm going to ask you some questions about your house.

1. In what year did you move to this house? __________

2. What year was this house built? __________

3. Has this house had a new addition, or been remodeled, renovated, or painted on the inside?

   [ ] YES ------------- CONTINUE
   [ ] NO ------------- SKIP TO QUESTION 4
   [ ] DK ------------- SKIP TO QUESTION 4

   3a. What was the most recent year that an addition was added or when the house was remodeled, renovated, or painted on the inside? __________

   3b. What kind of work was done?

4. Do you have a full basement or crawl space?

[ ] BASEMENT --------------- 4a. Is the basement finished or unfinished?

[ ] CRAWL SPACE

[ ] NEITHER   [ ] FINISHED

[ ] UNFINISHED

5. Were any rugs or carpets in this house purchased new within the last year?

[ ] YES --------
[ ] NO

5a. Is there a new rug or carpet in the room where we are collecting the air sample?

[ ] YES

[ ] NO

6. Do you have any large pieces of furniture, such as a couch, sofa, upholstered chair, or bed, that were purchased new within the last year?

[ ] YES -------- 6a. Is new furniture in the room where we are collecting the air sample?
[ ] NO

[ ] YES

[ ] NO

7. Is there a garage attached to this house?

[ ] YES

[ ] NO

8. How many windows were open for at least an hour during the past 24 hours? _______

EQUIPMENT AND APPLIANCES

Next I'm going to be asking about the kinds of equipment and appliances you have in this house.

9. First, I'd like to know about all the kinds of heating that you have.

Do you have…?

		HAVE
9.1	electric heat	
9.2	natural gas	
9.3	oil heat	

10. Do you have a ...?

(IF YES) 10a. Was it in use in the last 24 hours while we were collecting

the air sample?

		HAVE		USED IN LAST 24 HOURS
10.1	wood stove		10.1a	
10.2	kerosene heater		10.2a	
10.3	wood-burning fireplace		10.3a	
10.4	gas-burning fireplace		10.4a	

11. My next questions are about kitchen, ventilation, and laundry appliances. Again, I'll be asking whether you have each one and whether it was operating during the past 24 hours while we were collecting the air sample.

Do you have a…?

(IF YES) 11a. Was it operating during the past 24 hours?

		HAVE		OPERATING
11.1	gas oven		11.1a	
11.2	gas range (stove)		11.2a	
11.3	indoor grill		11.3a	
11.4	dish washer		11.4a	
11.5	attic or window fans		11.5a	
11.6	clothes washer in living area		11.6a	

12. My next questions are about home office equipment.

Do you have a … in the house.?

(IF YES) 12a. Was it operating during the past 24 hours?

		HAVE
12.1	computer printer	
12.2	fax machine	
12.3	photocopier	

	OPERATING
12.1a	
12.2a	
12.3a	

PRODUCTS

13. Now I'd like to ask about some products you may use at home. I'd like to know whether you or someone else has used each of these products in the house during the past two days? [Interviewer note: 24 hours of sampling and 24 hours preceding. You may ask participant if you can check cabinets to answer the "What kind did you use" question. For example, "do you mind if we just take a look?"]

Did you use ... during the past two days?

		USED IN PAST 2 DAYS		
13.1	Solid air freshener			
13.2	Spray air freshener			
13.3	Hair spray			
13.4	Spray antiperspirant			
13.5	Laundry detergent		13.5a	What kind did you use? ___________________
13.6	Dishwasher detergent		13.6a	What kind did you use? ___________________

13.7	Spray-on surface cleaner (e.g., 409, Fantastik, Lysol)		13.7a	What kind did you use? ___________________
13.8	Oven cleaner			
13.9	Glues or adhesives			
13.10	Furniture polish		13.10a	What kind did you use? ____________________
13.11	Toilet cleaner		13.11a	What kind did you use? ____________________
13.12	Tub or tile cleaner		13.12a	What kind did you use? ____________________
13.13	Paint thinner or stripper			
13.14	Bug killers or pesticide		13.14a	What kind did you use? ____________________
13.15	Carpet cleaners			
13.16	Spot removers for fabric			
13.17	Moth balls			
13.18	Fingernail polish			

14. Did you or someone else in the house bring home any dry cleaning in the past two days?

[ ] YES

[ ] NO

15. Does anyone who lives here smoke tobacco?

[ ] YES
[ ] NO

Now I'm going back to questions about your house.

17. Is there a business that operates in the house?

YES [ ] ------------ 17a. What kind of business is that?
____
NO [ ]

18. Is there a workshop or hobby area in the house?

YES [ ] ------------ 18a. What kind of activities are involved?__________________________
NO [ ]

19. Next I'd like to know about any times this house has ever been sprayed or treated for bugs, including ants, termites, roaches, or other insects. I'm interested in recent years and also all the years back to when you moved here. And I am interested in bug treatments by you or by anyone else, including a service or professional.

Has this house ever been treated for bugs by you or anyone else?

[ ] YES ---- CONTINUE
[ ] NO ---- SKIP TO 20
[ ] DK ---- SKIP TO 20

19a. Was it treated for bugs in the past year?

[ ] YES ---- CONTINUE
[ ] NO ---- SKIP TO 19e

[ ] DK ---- SKIP TO 19e

19b. What month or months was it treated for bugs during the past year?

____

19c. What kind of bugs was it treated for? ________________________

19d. What was it treated with?___________________________________

SKIP TO 19k

19e. What is the most recent year this house was treated for bugs? ___________

19f. What kind of bugs was it treated for that year? ________________________

19g. What was it treated with? ____________________________

19h. How many times was it treated that year? ____________________________

(19i-j deleted)

19k. What other years was this house treated for bugs?

[ ] NONE -------- SKIP TO 20

(OTHERWISE) --- CONTINUE

19L. What kind of bugs was it treated for in those years?

19m. What was it treated with?

19N. How many times a year was it treated in those years?

	k. YEAR(S)	L. KIND OF BUGS	m. TREATED WITH	n. HOW OFTEN
19.1				
19.2				
19.3				
19.4				
19.5				

20. Now let's talk about treatments for the lawn or area outside this house. In thinking about the area outside your house, include the lawn, foundation, shrubs, trees, flower or vegetable garden, and any place on your property. Again, please think about recent years and also all the years back to when you moved here. **Has the lawn or area outside this house ever been treated with an insecticide or herbicide or other chemicals to control bugs or weeds?** Include treatments by yourself or another member of the household, but for this question **do not** include services provided by a professional lawn care service.

[INTERVIEWER: DO NOT INCLUDE FERTILIZER OR LIME TREATMENTS.]

[PROMPT: I'll get to lawn service in a minute, but now I want to talk about treatments by you or another member of the household.]

[ ] YES - CONTINUE
[ ] NO - SKIP TO 21

[ ] DK - SKIP TO 21

20a. Was it treated during the past year?

[ ] YES - CONTINUE
[ ] NO - SKIP TO 20e

[ ] DK - SKIP TO 20e

20b. What month or months was it treated during the past year?
____

20c. What was it treated for? [E.G. WEEDS, TYPE OF BUGS...]
____

20d. What was it treated with?____

SKIP TO 20k

20e. What is the most recent year it was treated? _______

20f. What was it treated for? [E.G. WEEDS, TYPE OF BUGS...]

____

20g. What was it treated with? ____

20k. What other years was the lawn or area outside this house treated for weeds or bugs?

[ ] NONE   ----------- ---   SKIP TO 21

OTHERWISE   -----   CONTINUE

20L. What was it treated for in those years?

20m. What was it treated with?

	k. YEAR(S)	L. TREATED FOR	m. TREATED WITH
20.1			
20.2			
20.3			
20.4			

21. Have you ever used a professional lawn care service at this house?

[ ] YES - CONTINUE

[ ] NO - SKIP TO 22

[ ] DK - SKIP TO 22

21a. What is the most recent year you used a professional lawn care service? _______

21b. What was the first year you used a professional lawn care service at this house? _____

21c. Were there other years you used a professional lawn care service here?

[ ] YES - CONTINUE
[ ] NO - SKIP TO 22

[ ] DK - SKIP TO 22

21d. What year(s) was that?

	YEAR(S)
21.1	
21.2	
21.3	
21.4	

PETS

22. Do you have any pets?

[ ] YES -- CONTINUE
[ ] NO -- SKIP TO 24

Next I'd like to know about each pet that you have and whether they have been in this house during the past 24 hours.

Do you have any ....?

(IF YES) 22a. How many?

22b. Has it (have they) been in the house during the past 24 hours?

		a. HOW MANY	b. IN THE HOUSE PAST 24 HOURS
22.1	DOGS		
22.2	CATS		
22.3	OTHER [SPECIFY TYPES]		
22.4			

22.5			

[INTERVIEWER CHECK: ASK ONLY IF PARTICIPANT HAS A CAT OR DOG.]

23. Has [Have any of] your [cat(s)/dog(s)] been treated for fleas?

[ ] YES -----------23a. What type of treatment was that? ________________________
[ ] NO

23b. When was the most recent treatment? ______________________

PEOPLE IN THE HOUSE

Next I'd like to get some information to help us interpret the air sample. The amount of activity in the house affects the air sample. I'd like to ask about the number of people who were in the house during the past 24 hours while we were collecting the air sample and about how many hours they were sleeping or doing quiet or active activities.

24. How many people were in the house, including yourself, during the past 24 hours? ______

24a. How many hours were you away from home during the past 24 hours?

24b. ...at home sleeping?

24c. ...at home doing quiet activities like reading or watching TV?

24d. ...at home doing more active things like cooking or cleaning?

[REPEAT FOR EACH ADDITIONAL PERSON]

PERSON	HOURS DURING PAST 24 HRS.			
	a. AWAY	b. SLEEPING	c. HOME/QUIET	d. HOME/ACTIVE

24.1					
24.2					
24.3					
24.4					
24.5					

ROOMS OF THE HOUSE

My next questions are about the rooms in the living area of the house. I'd like to record the approximate size of each room and how much of the floor is covered by carpet or rugs. Let's start with this room.

(INTERVIEWER:

OBSERVE OR ASK. RECORD IN GRID BELOW. REPEAT FOR EACH ROOM)

25a. INTERVIEWER OBSERVE: About what size is the room? (IN FEET)

25b. INTERVIEWER OBSERVE: Is there a rug or carpet?

(IF NO, MARK GRID)

(IF YES) 25c. ASK: About how old is it?

25d. OBSERVE: Is it wall to wall?

(IF RUG IS NOT WALL-TO-WALL)

25e. OBSERVE: Does the rug cover more than half the room?

				RUG/CARPET		
	ROOM	a. SIZE (FT)	b. ***NO*** RUG	c. YRS OLD	d. WALL-TO-WALL	e. > 1/2 ROOM
25.1						
25.2						
25.3						
25.4						
25.5						
25.6						
25.7						
25.8						
25,9						
25.10						
25.11						

26. Do you know of any unusual things that have happened in this house, such as a fire or an oil or chemical spill or anything?

    [ ] YES -------------- 26a. What happened? ____
    [ ] NO

27. Thank you very much for your participation in the household exposure study. This particular study is currently funded through the year 2008, but the research team has a long-standing interest in chemicals and health and will continue to analyze data in future years. We would like to ask if you would give us your permission for members of our team to continue

studying this data for the next ten years. Would that be OK with you?

[ ] YES
[ ] NO

28. People like you -- who have let us come to your homes to collect samples, have made a very important contribution to helping us understand the connections between the environment and health. We really appreciate that. The research team would like to learn more about what it was like for you to participate in the study and to hear the results for your home. Would it be OK to contact you again to interview you about your feelings and experiences of participating in the study?

    [ ] YES

    [ ] NO

29. Would you like to receive the results of the new testing we completed here today?

    [ ] YES

    [ ] NO

30. Would you like to receive updates about findings from the study overall, as we publish them?

    [ ] YES

    [ ] NO

31. Finally, in a study like this, it is sometimes helpful to be able to talk again with people who participated – for example, to clarify some information. Would it be OK to contact you again, if necessary?

    [ ] YES

    [ ] NO

(THANK R FOR PARTICIPATION)

(STOP TIME: ____:____)

Interviewer signature:_______________________________
